# Supplementary material for: Reconciling grain growth and shear-coupled grain boundary migration
Source: Nat Commun. 2017 Nov 24;8:1764. doi: 10.1038/s41467-017-01889-3 (PMC5700957; doi:10.1038/s41467-017-01889-3)
Supplement: Supplementary file 1 — Supplementary Information [file 41467_2017_1889_MOESM1_ESM.pdf]

SUPPLEMENTARY NOTE 1: GRAIN ORIENTATIONS AND GB PROPERTIES

Supplementary Table 1 gives the lattice orientations of each of the grains in the simple, idealized microstructure simulation. The grain labels correspond to those given in Fig. 3a in the Primary Text. Grains that share a label in Fig. 3a are periodic duplicates. With reference to Fig. 3a in the Primary Text, $\hat{x}$ is the horizontal direction, $\hat{y}$ is vertical, and $\hat{z}$ is normal to the plane of Fig. 3a. Grains A and B have the same lattice orientation, but are bounded by different GBs.

Supplementary Table 2 gives the Σ value, lattice planes, misorientation, mobility, and shear-coupling factor β for each of the GBs in the idealized microstructure simulation. As with the grain labels in Supplementary Table 1, the GB labels correspond to those given in Fig. 3a in the Primary Text. Measurements of mobility and β were conducted using bicrystal simulations at 1200K and under a driving force Ψ of 4.4 meV/Å^3 [1]. Supplementary Figures 1 and 2 give the GB migration and shear displacement data that was used to calculate these GB properties. GBs that share a label are either periodic duplicates or are related by a symmetry operation. All four GBs are asymmetric tilt GBs. The most notable conclusion from Supplementary Table 2 is that all GBs have comparable energy and mobility.

Grains in Idealized Microstructure			
Label	$\hat{x}$	$\hat{y}$	$\hat{z}$
A	$[2\ \bar{1}\ \bar{1}]$	$[0\ 1\ \bar{1}]$	$[1\ 1\ 1]$
B	$[2\ \bar{1}\ \bar{1}]$	$[0\ 1\ \bar{1}]$	$[1\ 1\ 1]$
C	$[23\ \bar{2}2\ \bar{1}]$	$[7\ 8\ 1\bar{5}]$	$[1\ 1\ 1]$
D	$[\bar{2}2\ 23\ \bar{1}]$	$[\bar{8}\ \bar{7}\ 15]$	$[1\ 1\ 1]$

Supplementary Table 1. **Grain Orientations.** Crystallographic directions of grains A, B, C, and D relative to the $\hat{x}$, $\hat{y}$ and $\hat{z}$ directions of the simulation cell.

Grain Boundaries in Idealized Microstructure					
Label	GB Character	θ	γ (J/m^2)	M m/(GPa·s)	β
1	$\Sigma39[111](23\ \bar{2}2\ \bar{1})/(2\ \bar{1}\ \bar{1})$	32.2°	1.58	113	0.23
2	$\Sigma13[111](8\ 7\ 1\bar{5})/(0\ \bar{1}\ 1)$	27.8°	1.56	130	0.29
3	$\Sigma13[111](\bar{2}2\ 23\ \bar{1})/(2\ \bar{1}\ \bar{1})$	27.8°	1.60	115	0.23
4	$\Sigma39[111](\bar{7}\ \bar{8}\ 15)/(0\ \bar{1}\ 1)$	32.2°	1.56	117	0.28

Supplementary Table 2. **Grain Boundary Properties.** GB label, character (Σ, boundary planes, misorientation angle θ) and properties γ (at 0 K), mobility M (at 1200 K), and shear coupling parameter β (at 1200 K) for the grain boundaries in Fig. 3a of the Primary Text.

SUPPLEMENTARY NOTE 2: BICRYSTAL SIMULATIONS FOR GBS FROM THE IDEALIZED MICROSTRUCTURE

Two sets of bicrystal simulations were performed for each of the four unique high angle grain boundaries in the idealized microstructure simulation. The GB labeling is consistent with Fig. 3a of the Primary Text. The first set (Supplementary Figures 1 and 2) is analogous to Fig. 5 of the Primary Text (i.e., the ends of the cell were unconstrained). In each simulation, the temperature was 1200K ($0.85T_m$) and the driving force was 4.4 meV/Å^3. The second set of simulations (Supplementary Fig. 3) are analogous to Figs. 6 and 7 of the Primary Text (i.e., the top and bottom ends of the simulation cell were fixed).

In Supplementary Fig. 1, the GB position H and shear stress τ are given as a function of time. Under the applied driving force, all four GBs migrate at a constant velocity until they reach the bottom of the simulation cell. In this time, there is no accumulation of stress. In Supplementary Fig. 2, the shear displacement B is plotted over the GB position H; the slope of these lines is equivalent to the inverse shear-coupling parameter β^{-1}. Even at this high temperature, all four GBs exhibit conventional shear-coupling. These simulations were used to determine the GB mobilities and β values given in Supplementary Table 2.

In Supplementary Fig. 3, τ and H are plotted as a function of time for the fixed-end simulations. GBs 1-3, shown in Supplementary Figs. 3a-c, accumulate stress and stagnate, in analogy with Fig. 6 of the Primary Text. This implies a large gap between the lowest-E_i mode and the lowest-E_i mode of opposite sign and explains why Grain A in Fig. 3a of the Primary Text does not shrink. However, GB 4 (Supplementary Fig. 3d) migrates to the bottom of the simulation cell and exhibits an oscillatory stress, similar to that observed in Fig. 7 of the Primary Text. This shear-coupling switch-back facilitates GB migration and enables Grain B to shrink. It should **not** be concluded that GBs 1-3 will permanently stagnate in the fashion of the one-mode coupling model. Instead, it is expected that the applied constraints will substantially **slow** GB migration relative to the free-end simulations in Supplementary Fig. 1.

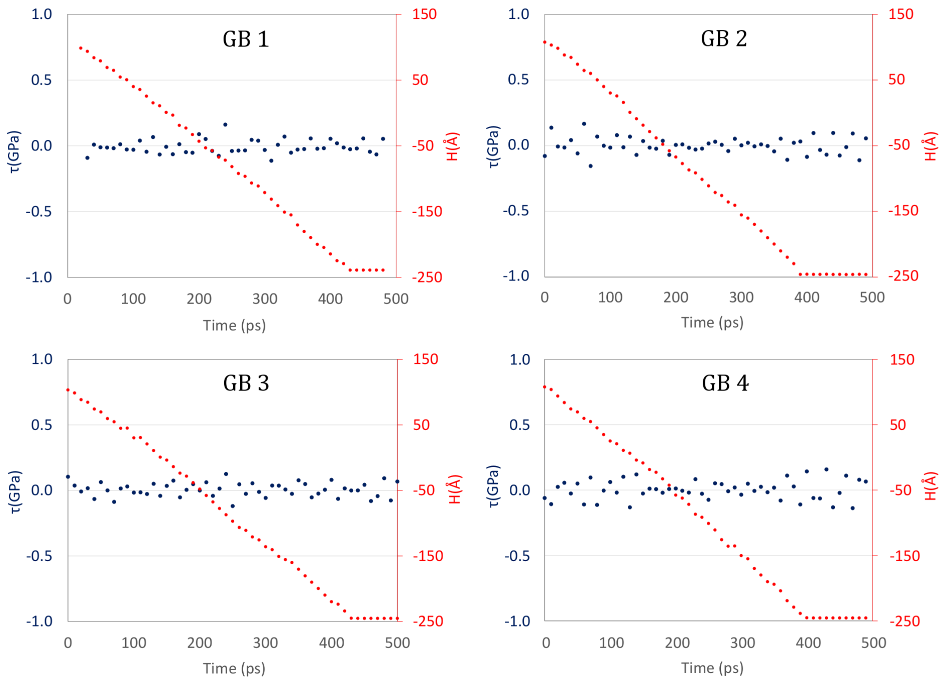

Supplementary Figure 1. **Unconstrained Bicrystal Simulations.** Shear stress τ and GB position H for the GBs labeled in Fig. 3a of the Primary Text. Simulations were performed under free-surface conditions. The dimensions of each simulation were approximately $490 \times 250 \times 244$Å.

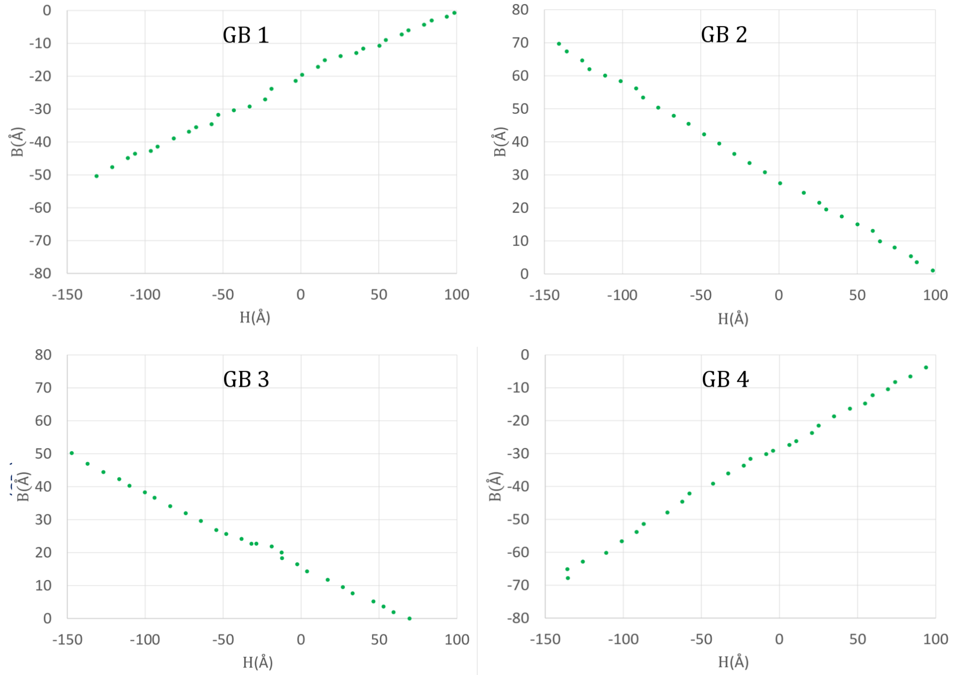

Supplementary Figure 2. **Shear-Coupling in Unconstrained Bicrystal Simulations.** B plotted against H from the same simulations as Fig. 1. The slopes of these curves correspond to the measured value of β^{-1} for each GB.

SUPPLEMENTARY NOTE 3: ASSUMPTIONS AND MATERIAL PROPERTIES

The model for disconnection nucleation discussed in the Primary Text assumes that the system is a bicrystal under periodic boundary conditions in the direction tangent to the GB. A disconnection is characterized by a Burgers vector

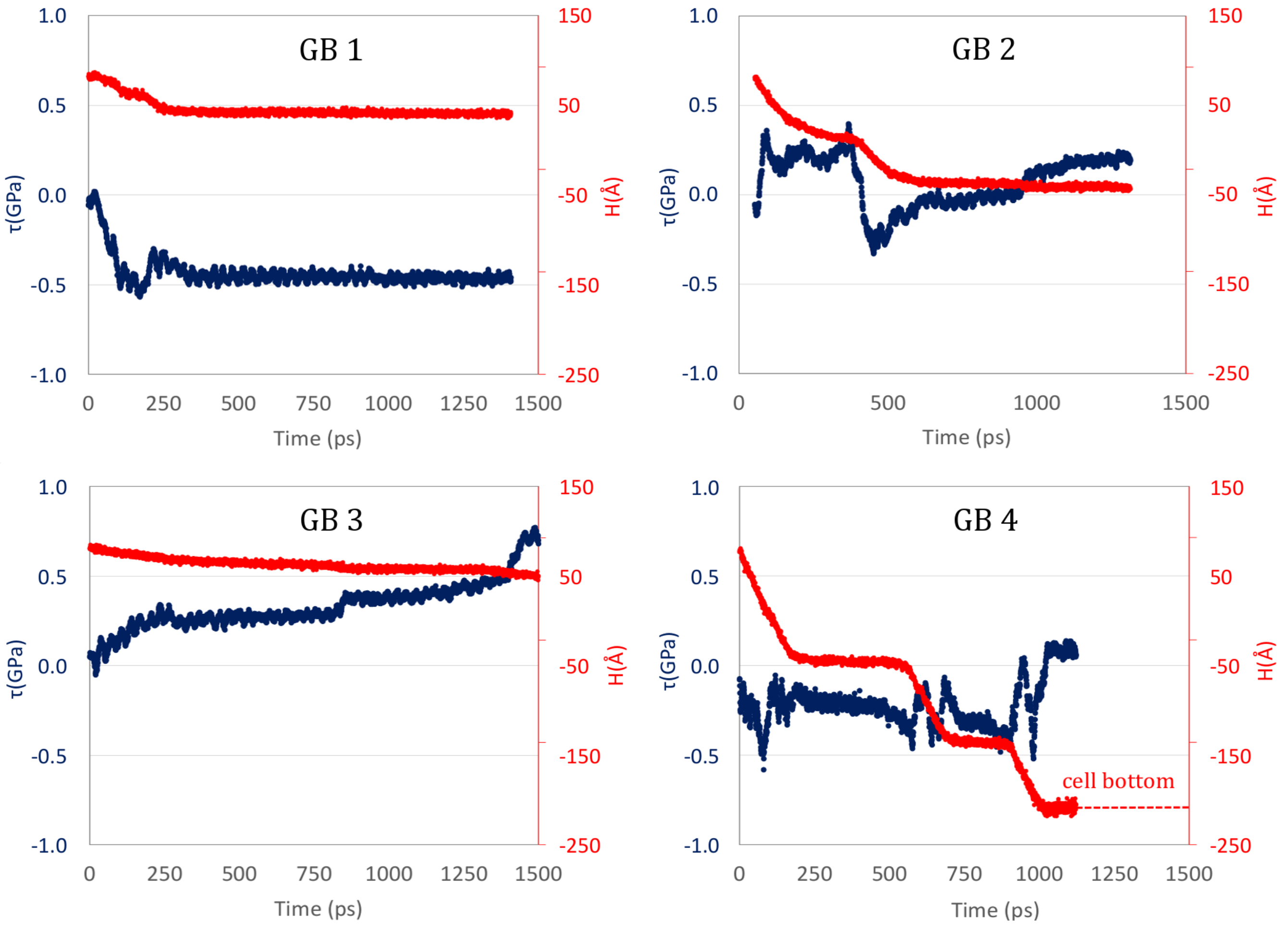

Supplementary Figure 3. **Constrained Bicrystals.** Shear stress τ and GB position H for the GBs labeled in Fig. 3a of the Primary Text. Simulations were performed under fixed-end conditions. The dimensions of each simulation were approximately $500 \times 250 \times 25$Å

$\mathbf{b}_i$ and GB step h_i, and a disconnection pair consists of two disconnections of opposite sign. When such a pair forms, the two disconnections may either move toward one another and mutually annihilate resulting in no GB migration, or separate until they annihilate (with their images across the periodic boundary) resulting in GB migration.

In the absence of an external driving force, the energy of the disconnection pair exhibits a maximum at a separation $\delta = w/2$ (note that the pair will annihilate at $\delta = 0$ or w due to the periodic boundary conditions). At this separation, the system energy is

$$E_i = 2\gamma_S|h_i| - \frac{G}{2\pi}\frac{1}{1-\nu}b_i^2 \ln\left[\sin\left(\frac{\pi\delta_0}{w}\right)\right]. \tag{1}$$

This expression appears as Eq. 11 in the Primary Text [2, 3]. An external stress τ provides a Peach-Koehler driving force [4] on the dislocation component of the disconnection, while a difference in energy-density between the two grains acts as a driving force on the step component of the disconnection. These two driving forces add $f_i = w(b\tau + \Psi h)/2$ to the energy E_i, above.

In our disconnection model, we assume that disconnection nucleation occurs infrequently compared with the time it takes for a disconnection pair to either migrate to the edge of the periodic cell, where it annihilates. The two primary consequences of this assumption are that (a) there is never more than one disconnection pair on the GB at a time and (b) we can approximate the GB migration rate in terms of the disconnection nucleation rate, neglecting the time required for disconnection migration to the end of the periodic cell. This is reasonable if the periodic cell is narrow (which is true for the simulations presented in this study) or the nucleation rate on a large GB is sufficiently large.

The calculations represented in Fig. 6 of the Primary Text require values of β, the GB mobility M, and the shear modulus G. β and M were computed directly from ($\Sigma 13[111]$ symmetric tilt) bicrystal simulations (see Fig. 5 of

the Primary Text). For this calculation, we use $G = 103$ GPa, which was calculated for the relevant crystallographic orientation from the elastic constants provided in the reference for the potential [5].

For all calculations of disconnection nucleation barriers, we used the shear modulus $G = 91.93$ GPa and the isotropic Poisson's ratio $\nu = 0.28$ (following the procedure of Hill [6] given the anisotropic elastic constants [5]). We calculated GB energies of 1.64J/m^2 and 1.79J/m^2 for the $\Sigma13$ and $\Sigma39$ GBs, respectively. In all cases, the dislocation core radius was assumed to be of the same magnitude as the Burgers vector. These values were used to infer the most favorable coupling modes of each GB. Similarly, a GB energy of 1.59J/m^2 was calculated for the $\Sigma13[001](510)$ symmetric tilt GB in Figs. 9a and 9b in the Primary Text. Figures 9c-e of the Primary Text, are independent of material properties.

SUPPLEMENTARY EQUATIONS

In the interest of brevity, this derivation was abridged in the Primary Text. This version is more explicit. Beginning with Eq. 3 in the Primary Text:

$$u_x(y,t) = \begin{cases} y\tan\gamma & y < H_0 \\ y\tan\gamma + (y - H_0)\beta & H_0 < y < H \\ y\tan\gamma + (H - H_0)\beta & y > H, \end{cases} \tag{2}$$

where $\beta = B/(H - H_0)$ (see Fig. 8 in the Primary Text). Since β is constant, this definition is equivalent to $\dot{B}/\dot{H}$. The lateral displacement at the top of the cell (Fig. 8d) is

$$D(t) = u_x(L,t) = L\gamma + \beta(H - H_0), \tag{3}$$

where we have made the small strain approximation $\tan\gamma \approx \gamma$. In the absence of body forces and for an isotropic elastic bicrystal, τ is constant throughout the bicrystal. Equilibrium and compatibility demands that at the GB $(H(t))$ [7],

$$\text{compatility} \rightarrow [\![\dot{u}_x]\!] + \dot{H}\left[\!\!\left[\frac{\partial u_x}{\partial y}\right]\!\!\right] = 0 \tag{4}$$

$$\text{equilibrium} \rightarrow [\![\tau]\!] = 0, \tag{5}$$

where $[\![g]\!]$ denotes the jump in a function g across the GB. Substituting $\tau = G\gamma$ (where G is the shear modulus),

$$\dot{D}(t) = \frac{L}{G}\dot{\tau}(t) + \beta\dot{H}. \tag{6}$$

The elastic driving force f_{El} on the GB is

$$f_{El} = -\left[\!\!\left[\frac{\partial u_x}{\partial y}\right]\!\!\right]\tau = \beta\tau. \tag{7}$$

If the GB velocity is proportional to the driving force (over-damped motion/linear response), then

$$\dot{H} = M(\Psi + \beta\tau), \tag{8}$$

where M is the GB mobility and $\Psi = \Psi_2 - \Psi_1$ includes any other contributions to the driving force (e.g. energy density difference between the two grains). For constant Ψ, we can combine Eqs. 5 and 7:

$$\dot{D}(t) = \frac{L}{G}\dot{\tau}(t) + M(\beta\Psi + \beta^2\tau). \tag{9}$$

For the special case where the disconnections are perfect steps ($\mathbf{b}_i = 0$), such that $\beta = 0$, then $\dot{H} = M\Psi$ and $\dot{D} = (L/G)\dot{\tau}$. GB migration is then decoupled from τ and D. In the remainder of the discussion, we implicitly assume that $\beta \neq 0$ (although this case presents no problem). We now consider two cases: (1) stress-controlled migration and (2) displacement-controlled migration.

Fixed Stress, $\tau = \tau^0$: First, we consider a constant stress or traction applied at the ends of the sample. From Eqs. 8 and 9, $\dot{D} = M(\beta\Psi + \beta^2\tau^0)$ and $\dot{H} = M(\Psi + \beta\tau^0)$. The GB migrates to the top of the cell and the top of the cell

displaces at a constant rate. The condition $\tau^0 = 0$ corresponds to the unconstrained bicrystal (Fig. 5); here, $\dot{H} = M\Psi$ and $\dot{D} = \beta M\Psi$, reflecting a commonly-used synthetic driving force/shear-coupled migration simulation approach [8].

Fixed displacement rate, $\dot{D} = \dot{D}^0$: Many studies of shear-coupled GB migration incorporate a fixed displacement rate $\dot{D}$ [9]. To model this, we rewrite Eq. 9 as $\dot{\tau} = (G/L)[\dot{D}^0 - M\beta(\Psi + \beta\tau)]$, and integrate:

$$\tau(t) = \frac{\dot{D}^0}{M\beta^2} - \frac{\Psi}{\beta} + \left(\tau^0 + \frac{\Psi}{\beta} - \frac{\dot{D}^0}{M\beta^2}\right) e^{-\frac{GM\beta^2}{L}t}. \tag{10}$$

Substituting this solution into Eq. 8 yields

$$\dot{H} = \frac{\dot{D}^0}{\beta} + M\left(\tau^0\beta + \psi - \frac{\dot{D}^0}{M\beta}\right) e^{-\frac{GM\beta^2}{L}t} \tag{11}$$

$$H(t) = H_0 + \frac{\dot{D}^0}{\beta}t - \frac{L}{G\beta^2}\left(\tau^0\beta + \psi - \frac{\dot{D}^0}{M\beta}\right)(e^{-\frac{GM\beta^2}{L}t} - 1) \tag{12}$$

At the $t \to \infty$ limit,

$$\tau^\infty = \frac{\dot{D}^0}{M\beta^2} - \frac{\Psi}{\beta} \qquad \dot{H}^\infty = \frac{\dot{D}^0}{\beta}. \tag{13}$$

If the displacement is fixed ($\dot{D} = \dot{D}^0$) and there is no driving force ($\Psi = 0$) or initial stress ($\tau^0 = 0$), we recover the simulation method of Cahn et al. [9]:

$$\tau(t) = \frac{\dot{D}^0}{M\beta^2}\left(1 - e^{-\frac{GM\beta^2}{L}t}\right) \tag{14}$$

$$\dot{H} = \frac{\dot{D}^0}{\beta}\left(1 - e^{-\frac{GM\beta^2}{L}t}\right), \tag{15}$$

which converge to a steady state stress and GB velocity at late times.

If, in analogy with the constrained simulations in Figs. 6 and 7, we set $\dot{D} = 0$ and $\tau^0 = 0$, we find

$$\tau^\infty = \frac{\dot{D}^0}{M\beta^2} - \frac{\Psi}{\beta} = -\frac{\Psi}{\beta} \tag{16}$$

$$\dot{H}^\infty = \frac{\dot{D}^0}{\beta} = 0 \tag{17}$$

$$H^\infty(t) = H_0 + \frac{\dot{D}^0}{\beta}t + \frac{L}{G\beta^2}\left(\tau^0\beta + \Psi - \frac{\dot{D}^0}{M\beta}\right) = \frac{L}{G\beta^2}\Psi. \tag{18}$$

The GB travels a finite distance before stopping with a steady-state stress consistent with the observations in Fig. 6 of the Primary Text. The time evolution of τ and H agrees with simulation results shown in Fig. 6d (solid, colored lines). Here, we have used the independently-measured values of β, G, M, and L (as described above).

SUPPLEMENTARY REFERENCES

---

[1] K. G. F. Jannsens, D. Olmsted, E. A. Holm, S. M. Foiles, S. J. Plimpton, P. M. Derlet, Computing the mobility of grain boundaries, Nature Materials 5 (2006) 124 – 127.

[2] A. H. King, D. Smith, The effects on grain-boundary processes of the steps in the boundary plane associated with the cores of grain-boundary dislocations, Acta Cryst. A36 (1980) 335–343.

[3] J. Han, V. Vitek, D. J. Srolovitz, To be published, 2017.

[4] M. Peach, J. Koehler, The forces exerted on dislocations and the stress fields produced by them, Physical Review 80 (1950) 436.

[5] Y. Mishin, D. Farkas, M. J. Mehl, D. A. Papaconstantopoulos, Interatomic potentials for monoatomic metals from experimental data and *ab initio* calculations, Phys. Rev. B 59 (1999) 3393–3407.

[6] R. Hill, The elastic behaviour of a crystalline aggregate, Proceedings of the Physical Society. Section A 65 (1952) 349.

[7] J. L. Ericksen, Introduction to the thermodynamics of solids (1998).

[8] E. R. Homer, S. M. Foiles, E. A. Holm, D. L. Olmsted, Phenomenology of shear-coupled grain boundary motion in symmetric tilt and general grain boundaries, Acta Materialia 61 (2013) 1048–1060.

[9] J. W. Cahn, Y. Mishin, A. Suzuki, Coupling grain boundary motion to shear deformation, Acta materialia 54 (2006) 4953–4975.
