# Supplementary material for: Reconciling grain growth and shear-coupled grain boundary migration
Source: Nat Commun. 2017 Nov 24;8:1764. doi: 10.1038/s41467-017-01889-3 (PMC5700957; doi:10.1038/s41467-017-01889-3)
Supplement: Supplementary file 3 — Description of Additional Supplementary Files [file 41467_2017_1889_MOESM3_ESM.docx]

**Description of Additional Supplementary Files**

File Name: Supplementary Movie 1

Description: Polycrystalline Grain Growth. Animation of the Molecular Dynamics polycrystalline grain growth simulation depicted in Figs. 1 and 2.

File Name: Supplementary Movie 2

Description: Idealized Microstructure Evolution. Animation of the Molecular Dynamics Idealized Microstructure simulation, with centrosymmetry coloring consistent with Fig. 3.

File Name: Supplementary Movie 3

Description: Idealized Microstructure Stress Generation. Animation of the Molecular Dynamics Idealized Microstructure simulation, with shear stress coloring consistent with Fig. 4a.

File Name: Supplementary Movie 4

Description: Idealized Microstructure Grain Rotation. Animation of the Molecular Dynamics Idealized Microstructure simulation, with grain rotation coloring consistent with Fig. 4b.
